# A Synthetic Model of the Mucosa for Oral Penetration Studies

**DOI:** 10.3390/membranes13120905

**Published:** 2023-12-12

**Authors:** Cristina Alonso, Meritxell Martí, Anderson Ramos, Ana Cristina Calpena, Beatriz Clares-Naveros, Luisa Coderch

**Affiliations:** 1 Surfactants and Nanobiotechnology Department, Institute of Advanced Chemical of Catalonia of CSIC, (IQAC-CSIC), Jordi Girona 18-26, 08034 Barcelona, Spain; cristina.alonso@iqac.csic.es (C.A.); meritxell.marti@iqac.csic.es (M.M.); anderson.ramos@iqac.csic.es (A.R.); 2 Department of Pharmacy and Pharmaceutical Technology, Universitat de Barcelona, Avda. Joan XXIII s/n, 08028 Barcelona, Spain; anacalpena@ub.edu; 3 Department of Pharmacy and Pharmaceutical Technology, Faculty of Pharmacy, University of Granada, 18071 Granada, Spain; beatrizclares@ugr.es; 4 Biosanitary Research Institute of Granada (ibs. GRANADA), Avda de Madrid 15, 18012 Granada, Spain

**Keywords:** synthetic membrane, mucosa membrane, waterproofing formulations, TMWL, in vitro permeation

## Abstract

The main objective of this study is the evaluation of the use of a synthetic membrane, Nuclepore, as a model for permeation studies through sublingual mucosa. The permeability of two types of membranes, porcine sublingual oral mucosa and a synthetic Nuclepore membrane, to water was compared. Moreover, the water permeability of membranes modified with waterproofing formulations was measured to study their ability to protect against the penetration of viruses, toxins, etc. A relatively high correlation (R^2^ 0.88) was obtained between the transmucosal water loss (TMWL) values of the artificial membrane and the mucosa. These results support the possible use of this synthetic membrane in the screening of the water permeability of formulations. In addition, studies of the permeation of different actives, drugs, and biocides through the two membranes were carried out, and these results were compared with their skin permeation data. The synthetic membrane does not seem to discern between compounds in terms of permeability. However, the permeation of caffeine through intact or modified membranes incorporating waterproofing formulations presents similar permeation profiles through the synthetic membrane and mucosa. The results from these assays should lend support to the use of this synthetic membrane when screening formulations to be applied in oral penetration studies.

## 1. Introduction

The oral mucosa consists of connective tissue known as the lamina propria, which is covered by a stratified squamous epithelium. The oral mucosa is covered by a stratified epithelium with a maturation pattern similar to that of the skin that provides a barrier against attack from endogenous or exogenous substances present in the oral cavity and prevents the loss of material from the underlying tissue. Here, morphological diversity can be found, ranging from regions of orthokeratinized mucosa to nonkeratinized mucosa [1].

Actives easily permeate the nasal or oral mucosa in contrast to their low penetration through the keratinized stratum corneum tissue of the skin. This is mainly due to the different lipid compositions and the packed structures they form. It has been shown that the main determinant of the barrier function of the skin is the lipid content of the epidermal stratum corneum rather than the thickness or number of corneocyte layers present [2,3]. Ceramides, fatty acids, and cholesterol are the major lipids in the skin stratum corneum that determine the permeability barrier [4,5].

It is generally accepted that the diffusion resistance of the oral/nasal mucosa is primarily associated with the intercellular lipids of the outer layers of the tissue. The nature of the intercellular material is therefore an important determinant of the permeability of the oral epithelium. Other physiological characteristics that distinguish mucosal tissues from skin, such as extensive vasculature, their moist surface, and the presence of mucus, should also be taken into account. Mucosal tissues are covered with mucus, containing antiseptic enzymes such as lysozyme, proteins such as lactoferrin, and anionic glycoproteins known as mucins [6]. At physiological pH, the mucus network carries a negative charge (due to the sialic acid and sulfate residues) which play a role in mucoadhesion [7]. Mucus and saliva play important roles during penetration and may contribute to the barrier layer of mucosal tissues [7,8,9].

The nonkeratinized regions of the oral mucosa are more permeable than the keratinized regions, making the floor of the mouth and underside of the tongue as well as the buccal regions more attractive for drug delivery. In fact, for more than a century, nitroglycerin has been delivered systemically by placement under the tongue to alleviate angina pain [10]. The buccal drug delivery route permits the delivery of much larger molecules than those that can permeate the skin. For transdermal delivery, the molecular weight cut-off is approximately 350 daltons. In a study of the diffusion of fluorescein-conjugated dextrans through the porcine buccal mucosa, the molecular weight cut-off was somewhere between 10,000 and 20,000 daltons [11]. This raises the possibility of delivering peptides and nucleic acids via this route.

Most drugs are administered orally as pills or liquids or by injection. However, the transdermal route of drug delivery has several advantages over the oral and parenteral routes. The mucosa is readily accessible; has a quick absorption; has a robust epithelium; and allows bypass of first-pass metabolism, thus avoiding degradation in the gastrointestinal tract [12,13]. However, some disadvantages can be mentioned: the interference of saliva, which can dilute the drug absorption; the environment of the oral cavity due to food consumption; and the permeability, which can be too low for certain drugs [12]. Recent advances in chemical, material, and engineering techniques bring great opportunities to improve intraoral system fabrication and applications owing to the high biocompatibility and functional diversity [13,14,15].

The suitability of a transmucosal or transdermal system is generally determined using a permeation study. Franz diffusion cells are widely used in an in vitro methodology to determine drug permeation through biological membranes. Permeation studies are considered the gold standard for assessing the delivery of drugs in a transmucosal or transdermal system. However, ethical and economic reasons pose a major problem to the availability and use of animals. Penetration studies play an essential role in the selection of drugs for dermal or transdermal application [16], and it is clear that in vivo experiments in humans are required since the goal of drug delivery is to treat humans. However, during the first stages of drug development, it is necessary to develop alternative assays using accessible and reproducible surrogates of in vivo human skin [17].

For transdermal diffusion, attempts have been made to create synthetic membranes to be used as human skin models for pharmaceutical and cosmetic formulations [5]. Recently, great effort has been put into developing artificial membranes as surrogates for human skin [18,19]. However, additional efforts must be made to mimic the complex composition of the lipid structure of the SC [20]. Recent studies investigated whether the inclusion of lanolin in a synthetic polycarbonate membrane (Nuclepore^®^ Cytiva, Buckinghamshire UK) enhances the membrane barrier and mimics mammal skin [21,22] as a suitable strategy to provide accurate modelling of the barrier properties of the skin.

Back to our issue, buccal mucosa permeability has also been deeply studied and extensively reviewed [12,23]. Buccal permeability models are essential to determine important permeation parameters, but not all models can adequately mimic the complex human buccal mucosa. In recent years, the use of in vivo models has been discouraged and significantly reduced due to the development of ex vivo oral mucosa models (derived from animals) or in vitro models (obtained from cell cultures) [23]. However, a standardized system that reproduces the properties of the oral mucosa and allows a rational synthesis of pharmaceutical formulations is highly desirable and not yet available [24]. Oral tissue from pigs is the most extensively used tissue for in vitro drug permeability studies [25]. Tissue-engineered oral mucosa equivalents have also been developed [26]. Artificial (synthetic) membranes have been applied in studies of drug permeability through the oral mucosa [27,28] but to a much lesser extent than in the case of skin [18]. The special ethical concern about the excessive use of animals to study systems that could be optimized by employing preliminary tests supports, regardless of the limitations, the focus on the use of artificial membranes at least in the first evaluation stage before in vivo experiments are planned.

Considering the difficulty of obtaining and working with oral mucosa, this work presents the possibility of using a synthetic membrane to perform preliminary experiments for mucosa penetration studies. The permeability to water of two types of membranes, porcine sublingual oral mucosa and the synthetic Nuclepore membrane, was compared. Water permeability was determined by assessing the transmucosal/transmembrane water loss parameter (TMWL) of the intact mucosa and Nuclepore membranes. Moreover, the permeability of water was measured for membranes modified with waterproofing formulations, which were studied for their ability to protect against the penetration of viruses, toxins, etc. [29,30]. In addition, the permeation of different actives, drugs, and biocides through the 34embranes (porcine mucosa and the synthetic membrane) was carried out, and the results were compared with their skin permeation data. Additionally, the permeation of the active substance caffeine through intact and modified membranes incorporating the waterproofing formulations was studied.

Thus, the main objective of this study is the evaluation of the use of a synthetic membrane, Nuclepore, as a model for permeation studies through sublingual mucosa. The comparative permeation of the two membranes are assessed for different actives as well as water and caffeine on the membranes modified with different waterproofing formulations. The results from these assays should lend support to the use of this synthetic membrane in the screening of formulations to be applied in oral penetration studies.

## 2. Materials and Methods

Three types of membranes were used in this study, a synthetic membrane and two biological membranes, porcine skin and porcine sublingual oral mucosa. Whatman^®^ Nuclepore ™ is a synthetic membrane (Cytiva, Buckinghamshire, UK) made of polycarbonate with a pore size of 0.05 µm that has been shown to have similar permeability to human mucous membranes. The biological membranes were a porcine sublingual mucosa and porcine skin. The porcine tongues with sublingual mucosa were supplied by the Faculty of Pharmacy of the University of Barcelona from the Hospital de Bellvitge campus according to the protocols of the ethics committee and with the supervision of the above institution. The mucous membrane was dermatomed to a thickness of 500–700 µm (Dermatome GA630, Aesculap, Tuttlingen, Germany), and portions of the sublingual oral mucosa were obtained to fit in the Franz diffusion cells. In addition, to determine the specific thickness, each mucosal portion was measured with a digital micrometer (MAHR, Göttingen, Germany). Porcine skin was obtained from the unboiled back of a Landrace large white pig (3 months old, weight around 40 kg) (supplied by the Department of Cardiology of the Clinic Hospital of Barcelona). Animal handling was approved by the Institutional Review Board and Ethics Committee of Institut d’Investigacions Biomèdiques August Pi i Sunyer (IDIBAPS), and the management of the animals conformed to the Guide for the Care and Use of Laboratory Animals. Porcine skin was dermatomed to a thickness of 500 ± 50 µm (Dermatome GA630, Aesculap, Germany) and stored at −20 °C until further use.

Caffeine (CAF), ketorolac tromethamine (KET), dexamethasone (DEX), and ivermectin (IVE) were purchased from Sigma (Sigma-Aldrich, St. Louis, MO, USA). Solutions of these four actives (1%) were prepared in ethanol (Merck, Darmstadt, Germany) for testing in the permeation study. Fungitrol (FUN) (Troy Chemical Iberia, Barcelona, Spain), permethrin (PER) (Tagros Chemical India, Tamil Nadu, India), and propiconazole (PRO) (Janssen, Beerse, Belgium) were also tested at a 1% concentration in ethanol (Merck, Darmstadt, Germany) in the permeation study.

### 2.1. Waterproofing Formulations

Five waterproofing formulations of each type of formulation (hydrophobic, hydrophilic, and liposomal), were studied. All ingredients were supplied by Sigma (Sigma-Aldrich, St. Louis, MO, USA) except when specified below. Preservation of the hydrophobic and hydrophilic formulations was performed in 100 mL of a clear solution containing methylparaben (0.18%), propylparaben (0.02%), propylene glycol (0.85%), and purified water.

(a) Hydrophobic formulations
  1. Tea tree oil mouthwash: glycerin (15%), sorbitol (4.5%), lauryl sulfate sodium (3%), ethanol (10%) (Merck, Darmstadt, Germany), and tea tree oil (1.5%) (Acofarma, Terrassa, Spain) in water.
  2. Semisolid anhydrous absorption base: lecithin (50%) in liquid Vaseline (50%).
  3. Lipophilic base MI: isopropyl myristate (10%) in Filant Vaseline.
  4. Lipophilic base TGCM: propylene glycol (10%) and medium-chain triglycerides (10%) in Filant Vaseline.
  5. Fluid anhydrous absorption base: soy lecithin (50%) and isopropyl palmitate (50%).
(b) Hydrophilic formulations
  6. Sodium carboxymethyl cellulose gel 4%: sodium carboxymethylcellulose (4%) and glycerin (10%) in water.
  7. Sodium hyaluronate gel 2%: sodium hyaluronate (2%) in water.
  8. Chitosan gel 2%: chitosan (2%) was dispersed in a lactic acid solution (1%) in water.
  9. Alginate gel 4%: alginate sodium (4%) was dispersed in water, and CaCl_2_ solution (4%) was added.
  10. PLX-CBP Gel: poloxamer in water (26%) was added to carbopol 940 to reach a final concentration of 1%.
(c) Liposomal formulations

The liposomal formulations tested in this work are listed in Table 1. The lipids (ceramide 3 and ceramide 6) were supplied by Evonik (Evonik, Essen, Germany), and phosphatidylcholine and hydrogenated phosphatidylcholine were supplied by Lipoid (Lipoid, Ludwigshafen, Germany). All liposomes were generated using the thin film hydration method. The lipids were dissolved in 3 mL of a mixture of chloroform:methanol (2:1, *v*/*v*) (Merck, Darmstadt, Germany). Then, the solvent was evaporated using a rotary evaporator at 50 °C and 100 rpm until a thin lipid film formed on the walls of the flask. The lipid film was then dried and hydrated using a 10% aqueous urea solution dissolved in PBS and repeatedly heated until a smooth white liposome mixture formed. The heating temperature used depended on the phase transition temperature of the components.

All these 16 formulations are evaluated in the aqueous permeability study in Section 3.1, and 3 of them (F3, F6, and F16) are applied to the sublingual oral mucosa and to the artificial membrane in the caffeine permeation study in Section 3.3.

### 2.2. Water Permeability Study by Determining Transmucosal/Transmembranal Water Loss (TMWL)

Two types of membranes were used in this study, an artificial versus a biological membrane, to determine the similarities and differences between them.

The barrier functions of the Nuclepore synthetic membrane and biological membranes (skin and mucosa) were evaluated by measuring the transmucosal/transmembranal water loss using a Tewameter^®^ TM300 (Courage-Khazaka, Cologne, Germany).

Transmucosal water loss (TMWL) measurements were carried out over the membrane that had been deposited in Franz static diffusion cells (FDC) (3 mL, 1.86 cm^2^, Lara-Spiral, Couternon, France). These cells consist of a donor chamber and a receptor chamber (3 mL volume) separated by a membrane, e.g., the skin, mucosa, or artificial membrane. The lower receptor compartment contained a solution of phosphate-buffered saline (pH = 7.6) (Sigma-Aldrich, St. Louis, MO, USA) and ethanol (Merck, Darmstadt, Germany) at a 1:1 ratio. The cells were placed in a thermostatic bath (Julabo, Seelbach, Germany) for acclimatization until reaching a surface membrane temperature of 32 ± 1 °C. Once the cells had stabilized for 1 h and reached the optimal temperature, TMWL measurements were performed in triplicate with a Tewameter^®^ TM300. These measurements were made before any the application of 70 µL of any formulation and reevaluated 1 h after application. In addition, one membrane without formulation application was used as a control.

### 2.3. In Vitro Permeation of Drugs and Biocides

The Nuclepore synthetic membrane, sublingual mucosa, and porcine skin dermatomed to a thickness of 500–700 µm were used to evaluate the permeation of drug/biocidal compounds. These studies were performed using a Franz vertical diffusion cell (Lara Spiral, Couternon, France) as described before. The receiving fluid (RF) used was PBS:EtOH (1:1) for drug permeation and EtOH:H_2_O (75:25) for biocide permeation, assuring sink conditions of the compounds. The recirculating bath system was at 43 °C to obtain a membrane surface temperature of 32 ± 1 °C. Parameters such as TMWL, humidity, and temperature of the skin and mucosal membranes were determined with a Tewameter TM 300 (Courage—Khazaka, Cologne, Germany) before the start of the permeation test.

Next, a pseudoinfinite dose (300 µL) of the drug or biocidal solution was applied to each Franz cell. Solutions were applied in triplicate to determine the kinetic parameters. The drug solution consisted of caffeine (CAF), ketorolac tromethamine (KET), dexamethasone (DEX), and ivermectin (IVE) dissolved in ethanol, each at a concentration of 1%. The permeation of three biocides, Fungitrol (FUN), propiconazole (PRO), and permethrin (PER), was also determined. The biocides were dissolved in ethanol, each at a concentration of 1%. All drug and biocide solutions are applied in the permeation kinetic study in Section 3.2, and only the caffeine solution is applied in the permeation test of the modified membranes in Section 3.3.

Samples (0.2 mL) were collected at different times (0 h, 0.5 h, 1 h, 2 h, and 4 h), and the same volume of receptor fluid was immediately added back for replacement. The active compounds were diluted in the appropriate graduated flasks and filtered through a 0.22 µm nylon filter (Cameo, Sigma-Aldrich, St. Louis, MO, USA) before being analyzed by high-resolution liquid chromatography with a diode array detector (HPLC-DAD).

The release of each compound was evaluated by determining the cumulative amount permeated (*Qn*, μg/cm^2^), which corresponds to the cumulative amount of the substance quantified in the receiving liquid per unit of surface area of the applied sample [31]. The equation used for this determination is as follows (1):

(1) $$Qn=\frac{Cn\times Vc+\sum_{i=1}^{n-1}\left(Ci\times Vs\right)}{A}$$

where *Qn* is the cumulative amount of active compound released at time *n* (μg/cm^2^); *Cn* is the concentration of active compound in the sample (μg/mL); *Vc* is the volume of the vertical diffusion cell (3 mL); $\sum_{i=1}^{n-1}\;Ci$ is the sum of the compound concentrations (µg/mL) determined in sampling intervals 1 to *n* − 1; *Vs* is the volume of the sample; and *A* is the surface area of application (1.86 cm^2^).

The experimental *Qn* and % compound release data were used to construct graphs showing permeation over time. The experimental permeation data for each compound (drug or biocide) over time best fit the absorption kinetics equation described by Mallandrich et al. [32]. In this step, we selected the best absorption model (zero order, first order, or Higuchi order) to represent the penetration kinetics of the compound through the different membranes. The model was determined with the nonlinear regression software STATGRAPHICS plus 5 (Statgraphics Technologies, Inc., The Plains, VA, USA), and the best equation was selected based on the highest correlation coefficient corrected for the number of degrees of freedom (R^2^ DoFs). Once the model was defined, it was possible to calculate the following parameters: flow (J, µg/cm^2^/h), permeability coefficient (Kp, cm·h^−1^), delay time (Tl, h), maximum concentration (Cmax, µg/cm^2^), maximum time (tmax, h), and area under the curve (AUC, µg/cm^2^/h).

Parameters were obtained from the graph. Fluxes (J, μg/cm^2^/h) from the tested compounds were calculated from the slope of the linear portion of the cumulative amounts permeated through the membrane per unit surface area versus time plot. The permeability coefficients (Kp, cm/h) were obtained by dividing the J by the initial drug/biocide concentration. And Cmax represented the maxima concentration permeated for each active substance.

### 2.4. HPLC/DAD Analytical Measurements

All analyses were performed with reverse-phase HPLC using an Agilent 1620 Infinity II LC System (Waldbronn, Germany) equipped with a quaternary pump (G7111B), autoinjector (G7167A), multicolumn thermostat (G7116A), and WR diode array detector (G7115A). The software used was OpenLab (version 2.2.0). Validation of the analytical procedures followed the guidelines developed by the International Conference on Harmonization (ICH) [33]. ICH guidelines were followed to obtain the calibration curve, limit of quantification (LoQ), and limit of detection (LoD). The HPLC-DAD analytical conditions and method for evaluating the seven active ingredients are detailed in Table 2.

### 2.5. Statistical Analysis

Statistical analysis was performed using STATGRAPHICS plus 5 software (Statgraphics Technologies, Inc., The Plains, VA, USA). The Kruskal—Wallis test is a nonparametric test that is used when the data do not have a normal distribution. This test was used to compare the permeation parameters of the different active compounds through the different membranes. Statistical significance was decided at the probability level of 0.05 (*p*). All results are expressed as the mean ± standard deviation (SD).

## 3. Results and Discussion

### 3.1. Water Permeability of Intact Membranes and Those with Protective Waterproofing Formulations

The use of biological membranes, both animal and human, is essential to deepen our knowledge of the skin barrier and the oral mucosa. However, due to ethical reasons or the complexity of obtaining, preserving, and reproducing biological membranes as well as the high costs of these methods, it is necessary to find synthetic membranes with a behavior similar to those of biological membranes and therefore eliminate the limitations of the previous methods. In addition, the use of artificial membranes will obviate the great intra- and interindividual variability.

Permeability is an indicator of membrane integrity/barrier function, which is assessed by measuring transepidermal water loss (TEWL) [34]. TEWL or, in mucosa studies, transmucosal water loss (TMWL), is a natural, noninvasive technique that can be used in both in vivo and in vitro assessments of skin or mucosa integrity. The water vapor flux across the stratum skin or mucosa surface is measured, which is an indicator of water diffusion through the stratum membrane and its barrier property (Section 2.2). Under stable ambient conditions, the human skin TEWL oscillates near 4–10 g/m^2^/h, depending on the skin area [18], and in mucosae, this value is near 60–80 g/m^2^/h. In the present work, water permeability was assessed to evaluate the similarities between the artificial membrane mucosa and the skin. As a consequence, a first screening phase was carried out to evaluate the TMWL values of 63 formulations on the synthetic membrane. The formulations with a better waterproofing effect than the synthetic membranes are those that were described in Section 2.1. The TMWL experimental data are shown in Table 3; this table also includes data from porcine sublingual mucosa.

Notably, both the artificial membrane and the sublingual mucosa had great permeability (80 g/h·m^2^ and 72 g/h·m^2^, respectively) compared to that of the skin (4–10 g/h·m^2^). The formulations are applied to the synthetic membrane and to the sublingual mucosa to see its waterproofing effect in order to obtain a barrier effect similar to that of the skin. From the results obtained, the hydrophobic formulations were taken as those that decreased the water permeability to a greater extent, reaching more than 90% [29]. Moreover, hydrophilic formulations have also been evaluated because they are easier to apply and more palatable; however, water permeability decreases by only 20 and 25% when they are applied to these membranes. The third type of formulation, the liposomal formulation, was chosen because liposomes can structure lipids in an aqueous environment. It is worth highlighting formulations 15 and 16 with two different types of ceramides (Cer3 and Cer6), whose application reduced water permeability by 40% [30].

**Figure 1:**
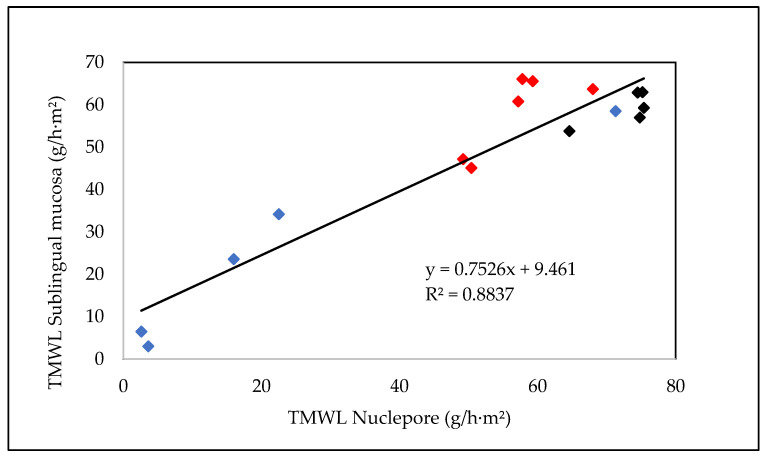
Correlation between the values of TMWL obtained with the Nuclepore artificial membrane and sublingual mucosa for the hydrophobic formulations (1, 2, 3, 4, and 5, in blue), hydrophilic formulations (6, 7, 8, 9, and 10, in black), and liposomal formulations (11, 12, 13, 14, 15, and 16, in red).

In this work, the application of several formulations to synthetic membranes was evaluated, mainly for their ability to modify the membranes’ permeability. This evaluation allows for the selection of membranes able to produce the desired effect, in this case, those membranes that become less permeable. Therefore, a correlation between the values of TMWL obtained with the Nuclepore artificial membrane and sublingual mucosa was evaluated for each formulation applied. Figure 1 correlates the TMWL data for the hydrophobic formulations (formulations 1, 2, 3, 4, and 5), hydrophilic formulations (6, 7, 8, 9, and 10), and liposomal formulations (11, 12, 13, 14, 15, and 16) on both types of membranes.

Despite the high water permeability of the Nuclepore membrane compared with the sublingual oral mucosa, it is important to note the high correlation between the TMWL data from the two membranes after the application of the external waterproofing formulation (Figure 1). This good relationship would support the possibility of using this synthetic membrane to screen a larger number of formulations, as previously performed to obtain these waterproofing formulations.

### 3.2. In Vitro Permeation Test of Drugs and Biocides

To study the characteristics of the synthetic artificial membrane and the sublingual mucosa membrane that allow the passage of active ingredients, four pharmaceutical drugs and three biocides were evaluated (Section 2.2). In addition, permeation through porcine skin was studied to be compared as a low-permeation membrane. In this sense, the behavior of the Nuclepore membrane and the two biological membranes was evaluated using actives with different molecular weights and lipophilicities.

Drug properties influence the permeation mechanism through the skin or mucosae. The physicochemical characteristics of a molecule are crucial to define the capacity of permeation across the skin layers. Molecules with small and large hydrophobic portions preferentially diffuse in lipid bilayers. Furthermore, large hydrophobic molecules have a low diffusion coefficient because of their large size. On the other hand, hydrophilic molecules preferentially permeate through the shunt pathway or via diffusion through the pores of the stratum corneum without interaction with lipid bilayers. Caffeine is a model hydrophilic compound that has been widely used in transdermal permeation studies. The permeation of caffeine and other more hydrophobic drugs with different physicochemical characteristics, such as ketorolac tromethamine, dexamethasone, and ivermectin, was evaluated with the different membranes as well as several biocides, such as Fungitrol, propiconazole, and permethrin.

Two different kinds of compounds were tested in the permeation test: drugs and biocides (Section 2.3, Section 2.4, Section 2.5). Drugs were selected based on their solubility and permeability characteristics. These two factors are directly related to the absorption process. Furthermore, each of them belongs to a different Biopharmaceutical Classification System (BCS) group [35]. The drugs evaluated were caffeine (CAF), ketorolac tromethamine (KET), dexamethasone (DEX), and ivermectin (IVE), each of which was dissolved in ethanol at a concentration of 1%. Permeation of the three biocides was also determined. The tested biocides were Fungitrol (FUN), propiconazole (PRO), and permethrin (PER), each of which was dissolved in ethanol at a concentration of 1%. The main physicochemical properties, such as lipophilicity (Log Ko/w) and molecular weight, important for permeability through keratinized tissues, including the skin and mucosa, are detailed in Table 4. On the one hand, the properties of these drugs differ greatly from one another due to their broad range of physicochemical characteristics, from very hydrophilic compounds with a low MW (such as caffeine) to compounds with high hydrophobicity and a large MW (such as ivermectin). On the other hand, the properties of the biocides Fungitrol and propiconazole were not so different, as these compounds have similar hydrophobicities and molecular weights, although permethrin has higher hydrophobicity and a larger molecular weight.

To effectively permeate through the skin membrane, compounds need to have a molecular weight below 500 Da, a partition coefficient (log Ko/w) of less than 5, and other physicochemical properties (including certain numbers of hydrogen bond donors or acceptors) [1,36]. The properties of the compound may change when it at an equilibrium and in contact with the biological membrane, which depends on the concentration of the drug and the composition of the medium in which it is dissolved. In this work, ethanol was used as the solvent. This solvent is a penetration enhancer that increases the flux of permeation [37]. However, fast evaporation of the organic solvent with infinite dosing would be expected to limit the enhancement of active diffusion.

**Table 4:** Partition coefficient (Log Ko/w), molecular weight (MW), and permeability coefficient (Kp, 10^−3^ cm/h) of different compounds through porcine skin (n = 4), sublingual porcine mucosa (n = 4), and the Nucleopore synthetic membranes (n = 4).

Compounds	Log Ko/w (pH 7.4)	Molecular Weight (MW)	Skin Kp (10^−3^ cm/h)	Sublingual Mucosa Kp (10^−3^ cm/h)	Nuclepore Kp (10^−3^ cm/h)
**Caffeine (CAF)**	−0.1 [38]	194.2 [38]	5.1 ± 4.1	39.2 ± 6.6	54.7 ± 11.2
**Ketorolac tromethamine (KET)**	2.3 [39]	376.4 [39]	2.2 ± 0.3	59.4 ± 0.6	67.5 ± 12.4
**Dexametasone (DEX)**	1.8 [40]	392.5 [40]	0.5 ± 0.3	25.7 ± 7.9	44.3 ± 7.0
**Ivermectin (IVE)**	5.8 [41]	875.1 [41]	0.3 ± 0.1	5.7 ± 3.1	41.1 ± 11.2
**Fungitrol (FUN)**	2.4 [42]	281.1 [42]	0.7 ± 0.4	4.0 ± 3.3	44.4 ± 11.9
**Propiconazole (PRO)**	3.5 [43]	342.2 [43]	0.2 ± 0.2	4.5 ± 3.6	40.8 ± 11.6
**Permethrin (PER)**	6.5 [44]	391.3 [44]	0.2 ± 0.1	2.7 ± 2.5	36.8 ± 17.0

Kinetic studies were conducted using manual vertical diffusion Franz cells and applying the active compounds to the skin, sublingual mucosa, and Nuclepore synthetic membrane. Studies were carried out in triplicate. The permeability coefficient (Kp, cm/h) results obtained for each active compound are reported in Table 4.

As expected, all compounds permeated very slowly through the skin due to the SC barrier. This skin barrier allowed small amounts of the compounds to reach the receptor fluid, as observed from the low-permeability coefficients. Caffeine, as the most hydrophilic compound with the lowest molecular weight, presented the highest permeability through the skin. The active permeability decreased as both the hydrophobicity and molecular weight increased. The penetration of the actives through the skin and sublingual mucosa was compared. As expected, the maximum penetration of the actives through the mucosa followed approximately the same order as the penetration through the skin. However, the synthetic membrane, even with its penetration profile that was always higher, did not seem to discern between compounds with different physicochemical properties. Very small differences in penetration through the synthetic membrane were found with the different compounds assayed. Therefore, it can be concluded that the Nuclepore membrane alone cannot be used as a model to determine the kinetic permeation of actives.

Nevertheless, the application of different formulations to the Nuclepore membrane make this membrane valuable due to it presenting similar TMWL behavior to that of the sublingual mucosa. Moreover, Nucleopore was also used to obtain alternative lanolin-based synthetic membranes for transdermal permeation and penetration assays evaluating drug delivery [21,22]. Therefore, after screening several formulations, the most efficient waterproofing formulations were applied to the sublingual mucosa and to the Nuclepore membrane to determine the penetration profile of an active compound such caffeine as a tracer.

### 3.3. In Vitro Permeation of Caffeine through the Modified Membranes

As in the previous TMWL assay, dermatomed porcine sublingual mucosa and split skin at a thickness of 500–700 µm as well as the synthetic Nuclepore membrane were used in this study. Kinetic diffusion studies with only caffeine on these virgin and modified membranes were performed as in the previous Section 3.2 using vertical diffusion cells as described in the Experimental Section 2.1 and Section 2.3, Section 2.4, Section 2.5.

Parameters such as TMWL, humidity, and temperature were determined for the skin and mucous membranes before starting the test with a Tewameter TM 300 (Courage + Khazaka, Cologne, Germany). Afterwards, 70 µL of the optimal formulations selected (formulations 3, 6, and 16) was deposited on each mucosal or synthetic membrane surface. These formulations had the lowest TMWL among each formulation type (hydrophobic, hydrophilic, and liposomal). After 1 h, the TMWL was remeasured, and these data were compared with the results already presented in Table 2. Finally, formulation 3 showed the greatest decrease in water permeability, followed by liposomal formulation F16 and, to a lesser extent, hydrophilic formulation F6, through both the sublingual mucosa and Nuclepore membrane.

**Figure 2:**
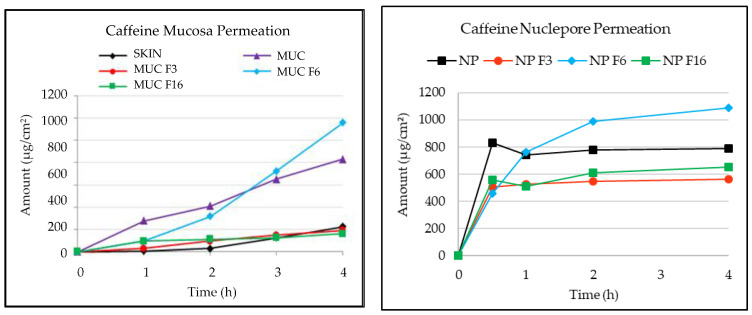
Cumulative amount of caffeine (µg/cm^2^) permeated through the skin (n = 4), sublingual mucosa (MUC, n = 4), and Nuclepore (NP) membrane free or modified by a waterproofing formulation (n = 4).

Caffeine was then deposited to determine the kinetic permeation of this tracer. In this experiment, 300 μL (infinite dose) of the 1% caffeine solution in ethanol was applied to each of the sublingual mucosa and Nuclepore membrane Franz cells in triplicate. Aliquots of receptor fluid were collected at different times and analyzed using high-performance liquid chromatography with a diode array detector (HPLC-DAD) as described in the Experimental Section. The results are expressed as the percent permeation (% permeation) over time and can be visualized in Figure 2 for each membrane.

Results for virgin membranes were already presented in the previous section but some parameters are repeated in this section to be specifically compared with the ones of the modified membranes (Figure 2 and Table 5). As expected, the permeation of caffeine through the skin was very low due to the presence of the stratum corneum, which acts as a barrier, reaching approximately 10% after 4 h (SKIN). The higher permeability of the sublingual mucosa was also demonstrated in this case by the higher capacity of caffeine delivery, with 40% release at 4 h (MUC). Moreover, the Nuclepore membrane (NP) allowed the highest release with no barrier to compound passage. Caffeine crossed this membrane rapidly, and the maximum permeation (50%) was obtained between 0.5 and 4 h. The permeation of caffeine through the differently modified membranes was compared. The maximum permeation was obtained for both the sublingual mucosa and Nuclepore membranes modified with hydrophilic formulation 6 (F6) (4% sodium carboxymethyl cellulose gel). It should be noted that this formulation seemed to increase the permeation of caffeine in a parallel manner between the two membranes, reaching 60% with mucosa (MUC F6) and 65% with the Nuclepore membrane (NP F6) at 4 h. The lowest permeation with both membranes was obtained after modification with hydrophobic formulation 3 (lipophilic base MI) and liposomal formulation 16 (Cer3Cer6 10%). These two formulations caused the membranes to become impermeable. In the case of the sublingual mucosa (MUC F3 and MUC F6), permeation was very similar to that of the skin (10% at 4 h), and in the case of the synthetic membrane, it was 40% for the two formulations (NP F3 and NP F6) at 4 h. Although the permeation percentages for the sublingual mucosa and the synthetic mucosa modified with the three formulations are not the same, a similar trend was noted. To confirm this observation, the parameters maximum concentration (Cmax) and permeability coefficient (Kp) were calculated and are shown in Table 5.

The maximum concentration (Cmax) and permeability (kp) values both confirmed the impermeability induced by the application of hydrophobic formulations F3 and F16 and the increase in permeability induced by the application of hydrophilic formulation F6 to the natural sublingual oral mucosa and synthetic mucosa. Therefore, it can be concluded that the synthetic Nuclepore membrane, which lacks lipids, does not have enough of a permeability barrier to discriminate between actives. However, this synthetic membrane can be used as a mucosa surrogate to determine the different behaviors of the applied formulations on the permeation of actives.

## 4. Conclusions

This work presented the possibility of using a synthetic membrane to perform previous trace experiments for mucosa permeation studies. The permeability to water and permeation of different actives through two types of membranes, porcine sublingual oral mucosa and a synthetic Nuclepore membrane, were compared.

A relatively high correlation was obtained between the values of TMWL with the artificial Nuclepore membrane and the sublingual mucosa. This result supports the possible use of this synthetic membrane in the screening of the water permeability of formulations. The permeation of the active through the synthetic artificial membrane and sublingual mucosa membrane indicates that the synthetic membrane did not discriminate between the compounds. This could be due to the lack of lipids in the composition of the synthetic membrane. However, the use of the synthetic membrane in permeation studies to determine the most efficient waterproofing formulation with a tracer active such caffeine presented similar results to the porcine sublingual oral mucosa. It should be noted that the most hydrophilic formulation seemed to increase the permeation of caffeine from the two membranes in a parallel manner, and the minimum amount of caffeine was obtained when both membranes were modified with hydrophobic formulation 3.

Therefore, it can be concluded that Nuclepore alone cannot be used as a model for the permeation studies of actives. However, the similar permeation to water and to caffeine, in the modified membranes, supports its use in preliminary screening studies to determine the mucosal waterproofing effect.

## 5. Patents

This work led to two patents:

Alonso, C.; Martí, M.; Coderch, L.; Calpena, A.C.; Mallandrich, M.; Pérez, L.; Clares, B.; Pérez, N. Lipophilic-based composition. N. Sol: EP23382737.7 (2023) N. Ref: ES1641.1822. CSIC, UB, UGR.

Alonso, C.; Martí, M.; Coderch, L.; Calpena, A.C.; Pérez, L.; Clares, B. Liposomal-based composition N. de Sol: EP23382651.0 (2023) N. Ref: ES1641.1823. CSIC, UB, UGR.

## Figures and Tables

**Table 1:** Compositions of the liposomal formulations.

	% Ingredient	Soy Phosphatidylcholine	Soy Hydrogenated Phosphatidylcholine	Ceramide 3	Ceramide 6	Cholesterol	Palmitic Acid
Formulation							
11	PC 10% (*w*/*w*)	10	--	--	--	--	--
12	HPC 10% (*w*/*w*)	--	10	--	--	--	--
13	Cer3 1% (*w*/*w*)	--	--	46.9	--	30.8	22.4
14	Cer3 10% (*w*/*w*)	--	--	46.8	--	31.6	23.0
15	Cer3Cer6 1% (*w*/*w*)	--	--	22.7	24.2	32.3	20.8
16	Cer3Cer6 10% (*w*/*w*)	--	--	23.1	23.5	30.9	22.5

**Table 2:** Method conditions used in the HPLC/DAD analysis of the different actives in the permeation study.

	Ketorolac Tromethamine	Caffeine	Dexamethasone	Ivermectin	Fungitrol	Propiconazole	Permethrin
Extractor solvent	PBS solution	Methanol LiChrosolv^®^	Methanol LiChrosolv^®^	Methanol LiChrosolv^®^	Methanol LiChrosolv^®^	Methanol LiChrosolv^®^	Methanol LiChrosolv^®^
Column	Lichrosphere^®^ 100RP-18, (250 × 4.6 mm, 5 µm)	Lichrosphere^®^ 100RP-18, (250 × 4.6 mm, 5 µm)	Lichosphere^®^ 100RP-18, (250 × 4.6 mm, 5 µm)	Lichrosphere^®^ 100RP-18, (250 × 4.6 mm, 5 µm)	Zorbax Eclipse XDB C18 (150 × 4.6 mm, 5 µm)	Zorbax Eclipse XDB C18 (150 × 4.6 mm, 5 µm)	Zorbax Eclipse XDB C18 (150 × 4.6 mm, 5 µm)
Wavelength (nm)	314	280	240	240	200	210	210
Injection volume (µL)	20	40	40	40	20	20	20
Mobile phase	NaH_2_PO_4_ (0.75 g/L + 0.5 mL H_3_PO_4_)/ CH_3_OH (340/660, *v*/*v*)	Methanol/water 60:40–90:10 (15′)–90:10 (15′)–60:40 (10′)	Methanol/water 60:40–90:10 (15′)–90:10 (15′)–60:40 (10′)	Methanol/water 60:40–90:10 (15′)–90:10 (15′)–60:40 (10′)	Acetonitrile/water 52/48–85/15 (10′) 85/15 (8′) 52/48 (5′)	Acetonitrile/water 52/48–85/15 (10′) 85/15 (8′) 52/48 (5′)	Acetonitrile/water 52/48–85/15 (10′) 85/15 (8′) 52/48 (5′)
Flux (mL/min)	1	1	1	1	1	1	1
Linear reg. eq. (R^2^)	A = 184,224 [KET] − 70,802 (0.9999)	A=51.512 [CAF]+2.002 (0.9993)	A = 5.352 [DS]+2.745 (0.9999)	A=42.000 [IVE] − 4.005 (0.9996)	A = 7.8218 [FUN] + 0.6291 (0.9986)	A = 41.302 [PRO] − 0.7125 (0.9987)	A = 34.733 [PER] + 0.0206 (0.9986)
LoD/LoQ (µg/mL)	0.10/0.28	0.82/2.49	0.23/0.70	0.55/1.66	1.09/3.31	0.37/1.11	0.49/1.49

**Table 3:** Transmembranal water loss from the Nuclepore synthetic membrane (n = 3), porcine sublingual mucosa (n = 3), and membranes after the application of the different formulations (n = 3).

Formulations	TMWL 1 h Nuclepore (g/h·m^2^)	TMWL 1 h Sublingual Mucosa (g/h·m^2^)
Nuclepore control	80.8	--
Sublingual mucosa control	--	72.4
(a) Hydrophobic formulations		
1 Tea tree mouthwash	71.3	58.5
2 Semisolid anhydrous absorption base	15.98	23.6
3 Lipophilic base MI	2.6	6.5
4 Lipophilic base TGCM	3.6	3.0
5 Fluid anhydrous absorption base	22.5	34.2
(b) Hydrophilic formulations		
6 Sodium carboxymethyl cellulose gel 4%	64.6	53.8
7 Sodium hyaluronate gel 2%	75.4	59.3
8 Chitosan gel 2%	75.2	63.0
9 Alginate gel 4%	74.5	62.9
10 Gel PLX-CBP	74.8	57.0
(c) Liposomal formulations		
11 PC 10%	57.8	66.1
12 HPC 10%	68.0	63.7
13 Cer3 1%	59.3	65.6
14 Cer3 10%	57.2	60.8
15 Cer3Cer6 1%	49.2	47.2
16 Cer3Cer6 10%	50.4	45.1

**Table 5:** Permeability coefficient (Kp, 10^−3^ cm/h) and maximum concentration of caffeine that permeated through the skin (n = 4), sublingual mucosa (n = 4), and Nuclepore membrane modified by waterproofing formulations (n = 4).

Parameter	SKIN	MUCOSA	MUCOSA F3	MUCOSA F6	MUCOSA F16	NUCLEPORE	NUCLEPORE F3	NUCLEPORE F6	NUCLEPORE F16
Permeability Coef. Kp (10^−3^ cm/h)	5.1 ± 4.1	39.2 ± 6.6	8.3 ± 2.5	49.3 ± 0.1	6.4 ± 0.9	54.7 ± 11.2	36.5	57.1	32.1
Maximal Conc. Cmax (µg/mL)	107.2 ± 57.7	514.5 ± 120.0	118.3 ± 34.8	719.7 ± 93.0	99.3 ± 8.5	507 ± 136	364 ± 247	673 ± 55	407 ± 131

## Data Availability

Data are contained within the article.
